# Statin use and all-cause mortality in people living with HIV: a systematic review and meta-analysis

**DOI:** 10.1186/s12879-018-3162-1

**Published:** 2018-06-05

**Authors:** Olalekan A. Uthman, Chidozie Nduka, Samuel I. Watson, Edward J. Mills, Andre P. Kengne, Shabbar S. Jaffar, Aileen Clarke, Tahereh Moradi, Anna-Mia Ekström, Richard Lilford

**Affiliations:** 1 Warwick-Centre for Applied Health Research and Delivery (WCAHRD), Warwick Medical School, University of Warwick, Coventry, CV4 7AL UK; 2 Division of Health Sciences, Warwick Medical School, University of Warwick, Coventry, UK; 3 McMaster University, Hamilton, Canada; 4 Non-Communicable Diseases Research Unit, South African Medical Research Council, Cape Town, South Africa; 5 Department of Medicine, University of Cape Town, Cape Town, South Africa; 6 Liverpool School of Tropical Medicine, Dept of International Public Health, Liverpool, UK; 7 Institute of Environmental Medicine, Division of Epidemiology, Karolinska Institutet Stockholm, Stockholm, Sweden; 8 Department of Public Health (IHCAR), Karolinska Institutet, Stockholm, Sweden; 9 Department of Infectious Diseases, Karolinska University Hospital, Stockholm, Sweden

**Keywords:** Statin, HIV, Mortality

## Abstract

**Background:**

It is unknown whether statin use among people living with HIV results in a reduction in all-cause mortality. We aimed to evaluate the effect of statin use on all-cause mortality among people living with HIV.

**Methods:**

We conducted comprehensive literature searches of Medline, Embase, CINAHL, the Cochrane Library, and cross-references up to April 2018. We included randomised, quasi-randomised trials and prospective cohort studies that examined the association between statin use and cardio-protective and mortality outcomes among people living with HIV. Two reviewers independently abstracted the data. Hazard ratios (HRs) were pooled using empirical Bayesian random-effect meta-analysis. A number of sensitivity analyses were conducted.

**Results:**

We included seven studies with a total of 35,708 participants. The percentage of participants on statins across the studies ranged from 8 to 35%. Where reported, the percentage of participants with hypertension ranged from 14 to 35% and 7 to 10% had been diagnosed with diabetes mellitus. Statin use was associated with a 33% reduction in all-cause mortality (pooled HR = 0.67, 95% Credible Interval 0.39 to 0.96). The probability that statin use conferred a moderate mortality benefit (i.e. decreased risk of mortality of at least 25%, HR ≤ 0.75) was 71.5%. Down-weighting and excluding the lower quality studies resulted in a more conservative estimate of the pooled HR.

**Conclusion:**

Statin use appears to confer moderate mortality benefits in people living with HIV.

**Electronic supplementary material:**

The online version of this article (10.1186/s12879-018-3162-1) contains supplementary material, which is available to authorized users.

## Background

Although life expectancy for people living with HIV has improved dramatically over the past two decades following the introduction of highly active antiretroviral therapy, mortality rates remain higher than in the general population [1, 2]. As life expectancy has increased, the incidence of non-communicable diseases (NCDs) including cardio-metabolic disorders has also disproportionately increased along with risky behaviours such as smoking, and has been identified as a major cause of excess mortality [3]. Dyslipidaemias are perhaps the most common and most studied cardio-metabolic disorders affecting people with HIV [4]. There are strong reasons to hypothesise that statins are effective in reducing cardiovascular events in people living with HIV. First, they are effective in many groups and among high-risk people who do not have HIV [5–17]. Second, they have been shown to reduce dyslipidaemia in HIV-infected people [18]. Third, they have been shown to act anti-inflammatory agent [19, 20] and improve surrogate markers for cardiovascular events, such as carotid intima-media thickness [21] and coronary artery plaque volume in people living with HIV [22].

Although studies of statins in HIV have evaluated subclinical CVD, none has evaluated associations between statin use and hard CVD endpoints. This includes nonfatal CVD events such as MI and stroke as well as CVD mortality. Thus, the logical next step would be to evaluate statin use and hard CVD endpoints for HIV+ persons. To date, no randomised trials have been published on this topic. However, analyses of hard CVD endpoints, including CVD mortality, may be underpowered due to insufficient data on hard CVD endpoints and/or cause-specific mortality in HIV+ cohorts and trial registries where statin use was assessed. As a result, this study sought to leverage the best currently available data and primarily evaluate overall mortality among HIV+ persons taking vs. not taking statins, with the evaluation of CVD mortality as an exploratory analysis. Evidence regarding the potential benefits of a particular intervention is often available from a variety of disparate sources. When considering the benefit of an intervention - particularly in the absence of any RCTs addressing the relevant question - that ‘real-world’ evidence from non-randomized studies should also be considered [23]. We aimed to examine whether statin use is associated with all-cause mortality using a systematic review and meta-analysis of prospective cohort studies.

## Methods

### Eligibility criteria

To be included, studies had to meet the following selection criteria:

- *Types of studies*: Randomised, quasi-randomised trials and prospective cohort studies.
- *Types of participants*: adult (> 18 years) people living with HIV (PLHIV) of either sex.
- *Types of intervention*: Any form of statin use regardless of indication, including but not limited to primary or secondary prevention of cardiovascular disease.
- *Types of comparator:* no statin or placebo
- *Types of outcome measures*: All-cause mortality.

### Information sources and search strategy

We conducted a thorough literature search to identify relevant studies. We searched electronic databases of Medline, CINAHL and Web of Science from 1980 to April 2018 without applying any language restriction. We searched for abstracts of relevant conference proceedings from the National Library of Medicine Gateway. In addition, the bibliographies of retrieved articles were examined for pertinent studies. The full Medline search strategy is shown in Additional file 1: Appendix S1.

### Study selection and data extraction

Two authors (OU and NC) evaluated the eligibility of studies obtained from the literature search using a predefined protocol and worked independently to scan all abstracts and obtain the full texts of each selected article. For each included study, details on design, population characteristics, intervention and outcome measures were extracted, and risk of bias was evaluated. Any discrepancies between the authors were resolved through discussion and involving a third author. Two authors (OU and NC) independently extracted data.

### Risk of bias assessment

We used the Cochrane tool Risk Of Bias In Non-randomised Studies - of Interventions (ROBINS-I) to assess the risk of bias of included studies (see Additional file 1: Appendix S2) [24]. We assessed risks of bias in the following seven domains, facilitated by consideration of pertinent “signalling” questions, bias due to: confounding, selection of participants, measurement of interventions, departures from intended interventions, missing data, measurement of outcomes, selection of reported results. Within each domain, we rated risk of bias as “low” (comparable to a well performed randomised trial), “moderate” (sound for an observational study), “high” (there are important problems), or “very high” (the study is too problematic to provide useful evidence). The judgements within each domain were carried forward to an overall risk of bias judgement across bias domains.

### Statistical analysis

For the main analysis, we performed Bayesian random-effects meta-analysis [25] with a prior based on expected heterogeneity to pool the hazard ratio (HR) estimates for the association between statin use and all-cause mortality among studies [26]. We selected random-effects meta-analysis on account of anticipated heterogeneity in study population and methodology [26]. Treatment effect measures, the hazard ratio and odds ratio were log transformed to reduce skewness. We combined hazard ratio (HR) and odds ratio (OR) in the meta-analysis. HR and OR can be interpreted similarly if the underlying assumption of a generally low event risk (< 20%) is true [27].

We performed a meta-regression analysis to explore the relationship between the following study-level factors and reported treatment effects: sample size, publication year, cohort follow-up period, percentage of statin use, percentage male, mean age and study location (Europe vs. North America). Given the low number of studies identified in the study univariate meta-regressions were estimated as opposed to multivariate. All models were estimated using STAN and R [28].

### Sensitivity analyses

A number of sensitivity analyses were conducted to examine the robustness of the results to study quality and modelling assumptions. First, we assigned ‘quality weights’ to the studies on the basis of the risk of bias assessment [29]. The quality weights can be interpreted as the proportion of variance of a studies results not attributable to bias. Two sets of weights were used: (i) studies at high risk of bias were assigned a weight of 20% and studies at a moderate/low risk were assigned a weight of 70%; (ii) the respective weights for high risk and low/moderate risk were 50 and 80%. Second, we re-estimated the model excluding studies deemed as being at high risk of bias (a quality weight of zero). Third, we examined the sensitivity of the results to the choice of prior distribution. We used an informative ‘sceptical’ prior distribution based on the principle that ‘Most clinically important interventions are likely to reduce the relative risk of all-cause mortality by about 10-20%.’ The sceptical prior specifies that there was only a 5% probability that the HR was less than 0.75 [30].

This systematic review was reported according to the Preferred Reporting Items for Systematic Reviews and Meta-analyses (PRISMA) guidelines [31] (Additional file 1: Appendix S3).

## Results

### Study selection and characteristics

**Fig. 1:**
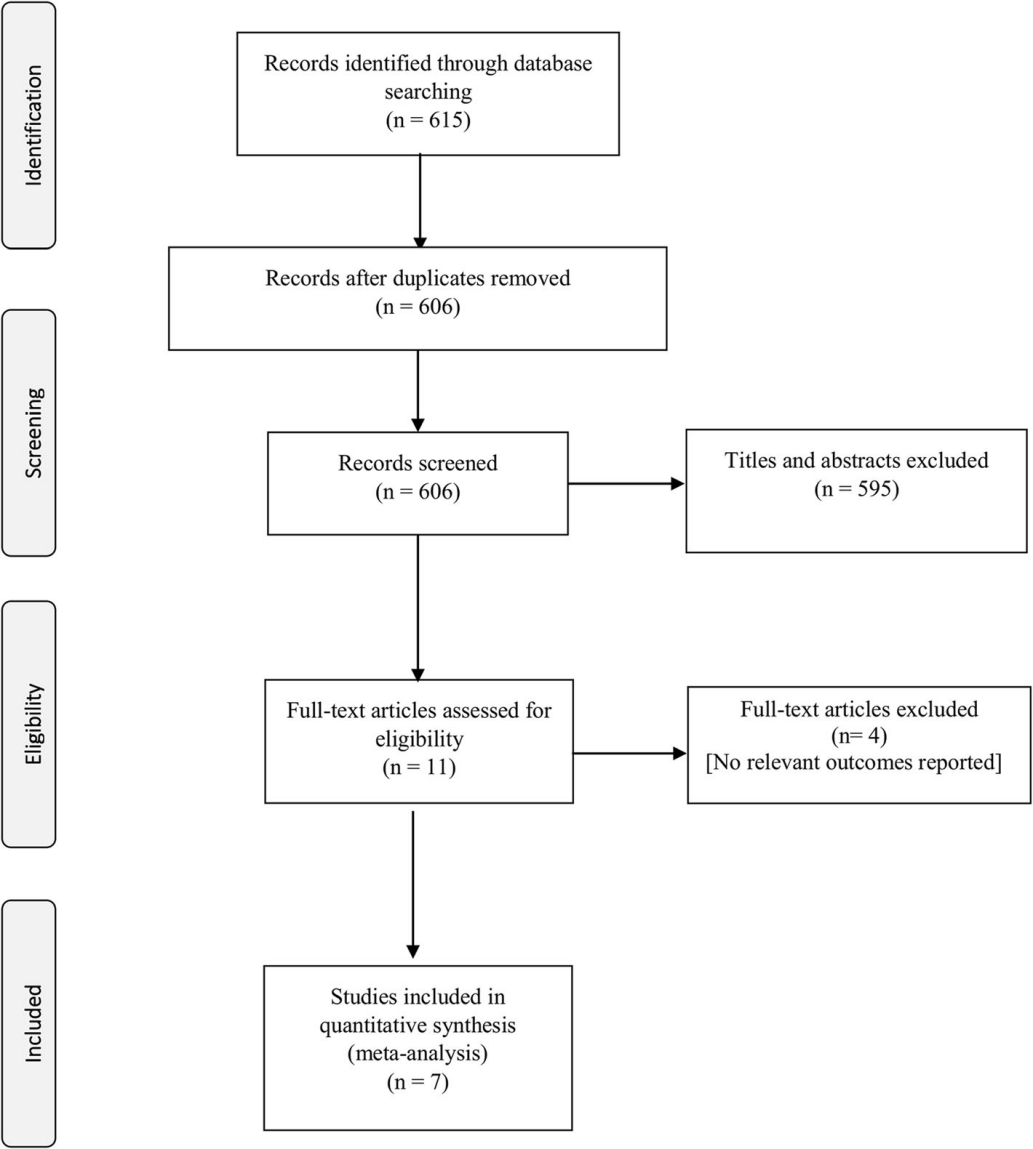
PRISMA Flow for study selection

The process of study selection is shown in Fig. 1. Overall, the literature searches of databases yielded 615 articles. After review of abstracts and titles, 11 articles were selected for critical reading. Four studies did not meet the inclusion criteria [32–35] as no relevant outcomes were reported. Seven studies with a total of 35,708 participants were included [36–42]. The characteristics of the included studies are shown in Table 1. The studies were conducted between 1995 and 2015 and published between 2011 and 2015. Five were reported as full-text journal articles [38–42], and two were presented as conference abstracts [36, 37]. All the studies were from high-income countries; four from the USA. The other studies were conducted in Denmark, France and Spain. The median number of participants was 1738 and ranged from 438 to 25,884. All the seven studies were cohort studies [36–42]. The mean age at entry of the participants ranged from 39 to 51 years and the proportions of males included in the studies ranged from 67% to as much as 98% (from US Veterans Affairs’ Clincial Case Registry). The percentage of participants on statins across the studies ranged from 8 to 35%. None of the study reported statin type. When reported, the percentage of participants with hypertension ranged from 14 to 35% and 7 to 10% had been diagnosed with diabetes mellitus. None of the studies reported rates of adherence to statins use.

**Table 1:** Summary Characteristics of the included studies

Study	Study period	Study design	Country	Setting/population	Indication	CVD risk	Male (%)	Age (mean/median)	Sample size	Statin therapy	Multivariate analysis (adjusted)
Moore et al., [40] 2011	1998 to 2009	Cohort	USA	Johns Hopkins HIV Clinical Cohort	Primary prevention	Antihypertensive use: 29.3%, total cholesterol: 166 (141–194) mg/dL	67.2	43 (36–49)	1538	Atorvastatin. Pravastatin, rosuvastatin [15.5%]	CD4, HIV-1 RNA, haemoglobin and cholesterol levels at the start of HAART, age, race, HIV risk group, prior use of ART, year of HAART start, NNRTI vs. PI-based ART, prior AIDS-defining illness, and viral hepatitis coinfection
^*^Drechsler et al., [36] 2013	1995 to 2009	Cohort	USA	Veteran Affairs’ Clinical Case Registry	Primary prevention	Smokers: 50%	98	46.8 (40.6–52.9)	25,884	Atorvastatin, rosuvastatin [35%]	Age, gender, race, HCV-co-infection, hypertension, smoking, BMI, CD4 strata, LDL-strata
^*^Knobel et al., [37] 2013	2002–2013	Cohort	Spain	HIV clinic in Barcelona	Primary prevention	Framingham score > 20%: 8.5%, ever smokers: 67%	72.2	42.09 (9.29)	733	Not reported [21%]	Baseline CD4 cell count, baseline viral load, undetectable viral load at follow-up, Framingham risk score, age, HIV transmission group, chronic liver disease, and smoking status
Overton et al., [41] 2013	2000–2013	Cohort	USA	Adult AIDS Clinical Trials Group Longitudinal Linked Randomized Trials (ALLRT)	Primary prevention	Framingham score > 10%: 10%, current smokers: 38%	83	39 (33–46)	3601	Not reported [13%]	Age, sex, race/ethnicity, intravenous drug history, history of coronary artery disease (CAD), hepatitis B coinfection, systolic BP, eGFR, glucose, current use of lipid-lowering drugs other than statins, HIV-1 RNA, CD4 count, current smoking, and waist-to-hip ratio.
Rasmussen et al., [42] 2015	1998–2009	Cohort	Denmark	Danish HIV Cohort Study (DHCS)	Primary prevention	Total cholesterol > 5 mmol/L: 28.3%	73.1	39.3 (33.0–46.3)	1738	Not reported [10%]	Age intervals (time-updated), gender, race, HIV-transmission group, hepatitis C status, calendar year of HAART initiation, AIDS defining illnesses prior to HAART, ART use before initiating HAART, CD4 cell count, viral load and cholesterol at HAART initiation.
Krask et al., [38] 2015	2000–2015	Cohort	USA	Nutrition For Healthy Living (NFHL)	Primary prevention	Framingham score: 6.5, hypertensive: 35%, diabetic: 7%, smokers: 47%, metabolic syndrome: 23%	68	44.3 (7.7)	438	Not reported [15%]	Race, HBV, HCV, LDL, CD4 cell count, age, smoking, statin duration
Lang et al., [39] 2015	2000–2009	Cohort	France	French Hospital Database on HIV (FHDH-ANRS CO4)	Primary prevention	Current smokers: 42.1%, hypertensive 13.7%, diabetics: 10.1%	88.9	50.5 (10)	1776	Not reported [8%]	Stepwise multivariable model using age, gender, HIV transmission group, current CD4 and CD8 T cell counts, CD4 T cell nadir, CD4/CD8 T cell ratio, CD4 T cell nadir/CD8 T cell ratio, plasma HIV-1 RNA level, AIDS status, the haemoglobin level, body mass index (BMI), smoking status, hypertension or use of antihypertensive treatment, diabetes or use of antidiabetic treatment, anti-HCV antibodies and HBs antigen status, non-AIDS malignancy (CIM-10 definition), liver failure, chronic kidney disease, cirrhosis, and pulmonary embolism.

*Conference abstracts

### Quality assessment of included studies

A summary of the risk of bias assessment in the included studies is shown in Additional file 1: Table S1. The risk of bias due to confounding was moderate all the seven studies. The bias in selection of participants was moderate in four studies [36, 38, 41, 42] and serious in three studies [37, 39, 40] For Knobel and colleagues study [37], the Framingham cardiovascular risk score above 20% was significantly higher among those on statin compared with those not on statins (21.4% versus 5.0%, RR = 2.95, 95% CI 2.22 to 3.92) and those on statin had significantly higher median total cholesterol (231 versus 178 mg/dL). Similarly, for Lang et al. [39], the proportion of participants with hypertension (29.7% versus 12.4%) and type 2 diabetes mellitus (21.7% versus 9.1%) was significantly higher among those exposed to statins compared with those not on statins at baseline. For Moore and colleagues study [40], the proportion of participants on antihypertensive medications was significantly higher among those on statin compared with those not on statins (46.3% versus 25.6%). The bias in measurement of interventions due to departures from intended interventions and due to missing data were moderate all the seven studies. The bias in measurement of outcomes was low in all studies, because the outcome of study was death ascertained via adequate record linkages [36–42]. The bias in selection of reported studies was rated serious in the two studies published as conference abstracts [36, 37] and moderate in the remaining five studies. Overall risk of bias was moderate in three studies and serious in four studies.

### Effects of statins on all-cause mortality

**Fig. 2:**
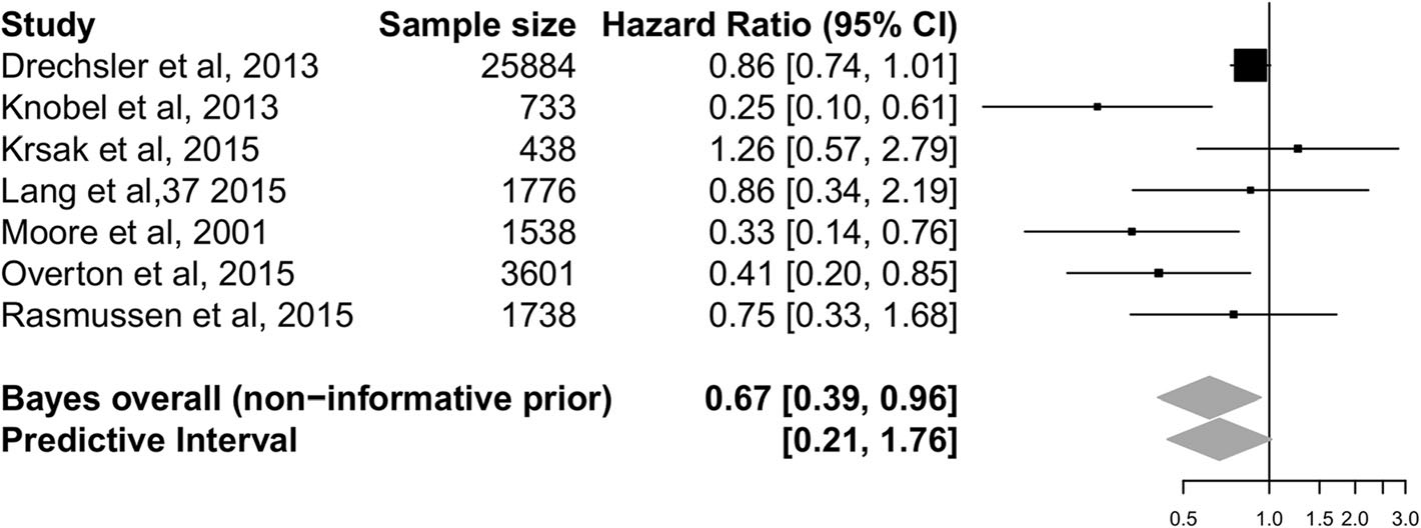
Forest plot of association between statins use and all-cause mortality

The Bayesian random-effects meta-analysis yielded a pooled HR of 0.67 (95% CrI 0.39 to 0.96) in the risk of all-cause mortality; a 33% reduction in absolute risk (Fig. 2). However, 95% prediction interval for the pooled HR contains values greater than 1 (0.21 to 1.76), which suggests that although on average statins seem to be effective in reducing all-cause mortality, not all future individual studies can be expected to show all-cause mortality benefits of statins.

Table 2 and Additional file 1: Table S2 reports the estimated pooled HRs from the sensitivity analyses. Down-weighting the lower quality studies resulted in a more conservative estimate of the pooled HR. The pooled HRs from the two weighting schemes were 0.82 (0.49, 1.35) and 0.76 (0.50, 1.13). Excluding low quality also resulted in a more conservative estimate of the pooled HR. The ‘sceptical’ prior resulted in a posterior mean estimate of the pooled HR of 0.88 (0.69, 1.14).

**Table 2:** Estimated pooled HRs from the sensitivity analyses

	Posterior mean (95% CrI)	Number of studies	I^2^	Probability HR < 1
Main analysis	0.67 (0.39, 0.96)	7	63%	97%
Down-weight low quality studies (20%/70%)^a^	0.82 (0.49, 1.35)	7	49%	80%
Down-weight low quality studies (50%/80%)^b^	0.76 (0.50, 1.13)	7	43%	92%
Exclude low quality studies	0.81(0.49, 1.37)	4	45%	81%
‘Sceptical’ prior	0.88 (0.69, 1.14)	7	62%	85%

In a series of meta-regression analyses, none of the study level factors were associated with treatment effect estimates (Table 3).

**Table 3:** Study level factors associated with treatment effect estimates, meta-regression analyses

Factor	Ratio of Hazard Ratio (95% Credible Interval)
Sample size (per 1000 people)	1.02 (0.97 to 1.06)
Publication year	1.20 (0.85 to 1.71)
Cohort follow-up period	1.16 (0.93 to 1.38)
Statin use (%)	1.01 (0.95 to 1.06)
Male (%)	1.02 (0.98 to 1.05)
Age (mean, years)	1.08 (0.98 to 1.18)
Europe vs. North America studies	0.94 (0.31 to 2.27)

Only two studies [37, 40] reported cardiovascular disease mortality as an outcome. The pooled HR from these two studies was 1.23 (0.52, 2.61). Only two studies [36, 41] reported cardiovascular disease event rates as on outcome. The pooled HR was 0.69 (0.37, 1.62).

## Discussion

### Main findings

To our knowledge, this systematic review and meta-analysis, comprising seven observational studies with more than 35,000 HIV-infected participants, is the first to examine the effect of statin therapy on all-cause mortality in people living with HIV. Overall, the findings support the expectation that statins confer mortality benefit. However, due to the limited evidence currently available, we can draw no conclusions as to effectiveness of statins on cardiovascular disease mortality and cardiovascular events. Nevertheless, the results of our meta-analysis usefully extend previously published meta-analyses of statin use in other high-risk groups such as the recent pivotal collaborative meta-analysis of individual participants’ data by the Cholesterol Treatment Trialists’ (CTT) [7] based on 174, 000 individuals, that reported reductions of approximately 10% in all-cause mortality for both women (risk ratio, 0.91; 99% CI 0.84–0.99) and men (risk ratio, 0.90, 99% 0.86–0.95). However, there has been conflicting literature about the association of statin use with all-cause mortality in other population [11, 13]. Ray and colleagues conducted a meta-analysis of 11 RCTs involving 65,2229 participants to examine the effect of statin use on all-cause mortality among intermediate to high-risk individuals without a history of CVD [11]. They found that the use of statins in this high-risk primary prevention setting was not associated with a statistically significant reduction (risk ratio, 0.91; 95% confidence interval, 0.83–1.01) in the risk of all-cause mortality [11]. Taylor and colleagues conducted Cochrane review to assess the effects, both harms and benefits, of statins in in adults with no restrictions on total, low density lipoprotein (LDL) or high-density lipoprotein (HDL) cholesterol levels, and where 10% or less had a history of CVD [13]. They included 18 RCTs involving 56,934 participants and found that all-cause mortality was reduced by statins (odds ratio 0.86, 95% confidence interval, 0.79 to 0.94) [13].

PLHIV are now living longer than because of effective treatment with antiretroviral therapy [43]. However, the increased life expectancy is now associated with increased prevalence of chronic conditions such as cardiovascular disease [43]. It has now been documented in the literature that PLHIV are at increased risk of developing CVD than non-HIV patients [43–45]. In addition, there is disparities in the quality of CVD care between PLHIV and uninfected adults [46]. Ladapo and colleagues found that “Physicians generally underused guideline-recommended cardiovascular care and were less likely to prescribe aspirin and statins to HIV-infected patients at increased risk-findings that may partially explain higher rates of adverse cardiovascular events among patients with HIV”. [46] Implementation of intensive lifestyle modification in PLHIV may help reduce CVD mortality and morbidity [43]. The frequency and consistency of clinical encounters with PLHIV may provide a unique opportunity to provide them with continuous assistance to help behaviour changes to prevent CVD risk [43]. Given the crucial need for prevention of cardiovascular disease in PLHIV, there is a need for a multi-morbidity trials to definitively assess the efficacy of statins as a primary prevention strategy for cardiovascular disease in this at-risk population [47, 48], especially in resource-limited settings that bear the highest burden of HIV. Furthermore, low income settings are now experiencing an epidemiological transition from infectious diseases to chronic diseases, [49] as a result of dramatic changes in diet and lifestyle. The epidemiological transition in LMICs is happening over a shorter time frame than that experienced historically by high-income countries [50].

### Study limitations and strengths

Strengths of this study include the comprehensive searches of databases to ensure that all relevant, published studies were identified. We used a Bayesian approach that allows us to utilize informative prior information. The main limitation of our study is that the nature of the literature is entirely observational. Statistical adjustment cannot exclude existing confounding, such as confounding by indication or variations in other patient or clinician level variability that might be independently associated with the outcome. Counter-intuitively, there is some evidence that statin may increase risk of CVD mortality, but perhaps not surprising because it likely reflects some confounding by therapy indication - people prescribed statins may be more likely to have pre-existing CVD or be at greater risk for CVD, and this risk is unlikely to be fully accounted for through multivariable adjustment. Thus, factors leading to these people being prescribed statins may be responsible for their apparently elevated CVD-related mortality rather than their actual use of statins. Statins are a large constellation with different results depending upon the type and dose of statin therapy. As these data were not reported, it was not possible to conduct sensitivity analyses to stratify by different types and doses of statins. In addition, the pooled association should be interpreted with caution because it is derived from observational studies. Our study found high *I*^*2*^ values as measures of heterogeneity. However, it is worth noting that the *I*^*2*^ measurement offers inflated estimates when dealing with non-comparative studies. Meta-regression analyses are prone to ecological fallacy (aggregate bias) and may have low power to detect an association [51–53]. We did not conduct tests for publication bias because we included observational studies and we are aware that many patients outside these studies receive statin treatment, so there is, by definition, publication bias. Finally, the fact that most included studies were observational may explain a larger treatment effect observed in our analysis than that observed among RCTs in non-HIV patients [7].

## Conclusions

In summary, based on pooled data on 35,708 HIV-infected participants from seven observational studies, we observed that statin therapy may have an important mortality benefit in people living with HIV, accounting for an estimated 33% reduction in all-cause mortality. While awaiting a definitive answer from on-going trials and long-term observational studies about the benefits of statin as a primary prevention for cardiovascular disease and all-cause mortality, our findings are timely and reassuring. They reinforce the notion that lowering lipid levels is likely to be associated with a reduction in all-cause mortality in people living with HIV as it is in other high-risk groups.

## Additional file

Additional file 1: **Appendix S1.** Medline Search Strategy. **Appendix S2.** Risk bias assessment. **Appendix S3.** PRISMA checklist. **Table S1.** Risk-of-bias assessment of included studies. **Table S2.** Estimated pooled HRs from the sensitivity analyses. (DOCX 41 kb)
