# Supplementary material for: Statin use and all-cause mortality in people living with HIV: a systematic review and meta-analysis
Source: BMC Infect Dis. 2018 Jun 5;18:258. doi: 10.1186/s12879-018-3162-1 (PMC5987595; doi:10.1186/s12879-018-3162-1)
Supplement: Supplementary file 1 — Appendix S1. Medline Search Strategy. Appendix S2. Risk bias assessment. Appendix S3. PRISMA checklist. Table S1. Risk-of-bias assessment of included studies. Table S2. Estimated pooled HRs from the sensitivity analyses. (DOCX 41 kb) [file 12879_2018_3162_MOESM1_ESM.docx]

**Additional file 1: DIGITAL CONTENT**

Contents

[Appendix 1: Medline Search Strategy 1](#_Toc514337332)

[Appendix 2: Risk bias assessment 2](#_Toc514337333)

[Appendix 3: PRISMA checklist 3](#_Toc514337334)

[eTable 1: Risk-of-bias assessment of included studies 5](#_Toc514337335)

[eTable 2: Estimated pooled HRs from the sensitivity analyses 6](#_Toc514337336)

## Appendix S1: Medline Search Strategy

| 1. (hiv or hiv or hiv-1 or hiv-2).mp.  2 human immunodeficiency virus.mp.  3 human immunedeficiency virus.mp.  4 human immune-deficiency virus.mp.  5 hiv infections.mp.  6 aids.ti,ab.  7 acquired immune-deficiency syndrome.mp.  8 acquired immunedeficiency syndrome.mp.  9 acquired immunodeficiency syndrome.mp.  10 acquired immuno-deficiency syndrome.mp.  11 (HAART or highly active anti?retroviral therapy or highly active anti retroviral therapy).mp.  12 Antiretroviral Therapy.mp.  13 retroviral*.mp.  14 Antiviral Agents.mp.  15 human immunodeficiency.mp.  16 antiretroviral*.mp.  17 exp hiv infections/  18 exp human immunodeficiency virus/  19 exp acquired immune-deficiency syndrome/  20 exp acquired immunodeficiency syndrome/  21 exp acquired immuno-deficiency syndrome/  22 exp hiv/  23 exp hiv-1/  24 exp hiv-2/  25 exp HIV SERONEGATIVITY/  26 exp HIV SEROPOSITIVITY/  27 exp HIV SEROPREVALENCE/  28 exp HIV ANTIBODIES/  29 exp ANTIRETROVIRAL THERAPY, HIGHLY ACTIVE/  30 exp Antiretroviral Therapy/  31 or/1-30  32 exp Hydroxymethylglutaryl-CoA Reductase Inhibitors/  33 (statin or statins).mp.  34 atorvastatin.mp.  35 cerivastatin.mp.  36 fluvastatin.mp.  37 lovastatin.mp.  38 pravastatin.mp.  39 simvastatin.mp.  40 lipitor.mp.  41 baycol.mp.  42 lescol.mp.  43 mevacor.mp.  44 altocor.mp.  45 pravachol.mp.  46 lipostat.mp.  47 zocor.mp.  48 mevinolin.mp.  49 compactin.mp.  50 fluindostatin.mp.  51 rosuvastatin.mp.  52 or/32-51  53 31 and 52  54 limit 53 to human |
| --- |

## Appendix S2: Risk bias assessment

| **Domains of bias**  *Pre-intervention (baseline)*  1. Bias due to confounding  2. Bias in selection of participants into the study  *At intervention*  3. Bias in measurement of interventions  *Post-intervention*  4. Bias due to departures from intended interventions  5. Bias due to missing data  6. Bias in measurement of outcomes  7. Bias in selection of the reported result  **Judgment about risk of bias (RoB) for each domain**   - Low RoB: the study is comparable to a well-performed randomized trial with regard to this domain - Moderate RoB: the study is sound with regard to this domain, but cannot be considered comparable to a well-performed randomized trial - Serious RoB: the study has some important problems in this domain - Critical RoB: the study is too problematic in this domain to provide any useful evidence on the effects of intervention   **Overall RoB judgment for each study**   - Low RoB: the study is judged to be at low RoB for all domains - Moderate RoB: the study is judged to be at low or moderate RoB for all domains, and moderate in at least one domain - Serious RoB: the study is judged to be at serious RoB in at least one domain, but not at critical RoB in any domain - Critical RoB: the study is judged to be at critical RoB in at least one domain |
| --- |

## Appendix S3: PRISMA checklist

| **Section/topic** | **#** | **Checklist item** | **Reported on page #** |
| --- | --- | --- | --- |
| **TITLE** | | |  |
| Title | 1 | Identify the report as a systematic review, meta-analysis, or both. | 1 |
| **ABSTRACT** | | |  |
| Structured summary | 2 | Provide a structured summary including, as applicable: background; objectives; data sources; study eligibility criteria, participants, and interventions; study appraisal and synthesis methods; results; limitations; conclusions and implications of key findings; systematic review registration number. | 2 |
| **INTRODUCTION** | | |  |
| Rationale | 3 | Describe the rationale for the review in the context of what is already known. | 3 |
| Objectives | 4 | Provide an explicit statement of questions being addressed with reference to participants, interventions, comparisons, outcomes, and study design (PICOS). | 3 |
| **METHODS** | | |  |
| Protocol and registration | 5 | Indicate if a review protocol exists, if and where it can be accessed (e.g., Web address), and, if available, provide registration information including registration number. | - |
| Eligibility criteria | 6 | Specify study characteristics (e.g., PICOS, length of follow-up) and report characteristics (e.g., years considered, language, publication status) used as criteria for eligibility, giving rationale. | 5 |
| Information sources | 7 | Describe all information sources (e.g., databases with dates of coverage, contact with study authors to identify additional studies) in the search and date last searched. | 4 |
| Search | 8 | Present full electronic search strategy for at least one database, including any limits used, such that it could be repeated. | 4 |
| Study selection | 9 | State the process for selecting studies (i.e., screening, eligibility, included in systematic review, and, if applicable, included in the meta-analysis). | 5 |
| Data collection process | 10 | Describe method of data extraction from reports (e.g., piloted forms, independently, in duplicate) and any processes for obtaining and confirming data from investigators. | 5 |
| Data items | 11 | List and define all variables for which data were sought (e.g., PICOS, funding sources) and any assumptions and simplifications made. | 5 |
| Risk of bias in individual studies | 12 | Describe methods used for assessing risk of bias of individual studies (including specification of whether this was done at the study or outcome level), and how this information is to be used in any data synthesis. | 6 |
| Summary measures | 13 | State the principal summary measures (e.g., risk ratio, difference in means). | 6 |
| Synthesis of results | 14 | Describe the methods of handling data and combining results of studies, if done, including measures of consistency (e.g., I^2^) for each meta-analysis. | 6-7 |
| Risk of bias across studies | 15 | Specify any assessment of risk of bias that may affect the cumulative evidence (e.g., publication bias, selective reporting within studies). | 5 |
| Additional analyses | 16 | Describe methods of additional analyses (e.g., sensitivity or subgroup analyses, meta-regression), if done, indicating which were pre-specified. | 7 |
| **RESULTS** | | |  |
| Study selection | 17 | Give numbers of studies screened, assessed for eligibility, and included in the review, with reasons for exclusions at each stage, ideally with a flow diagram. | 7 |
| Study characteristics | 18 | For each study, present characteristics for which data were extracted (e.g., study size, PICOS, follow-up period) and provide the citations. | 7-8 |
| Risk of bias within studies | 19 | Present data on risk of bias of each study and, if available, any outcome level assessment (see item 12). | 8-9 |
| Results of individual studies | 20 | For all outcomes considered (benefits or harms), present, for each study: (a) simple summary data for each intervention group (b) effect estimates and confidence intervals, ideally with a forest plot. | 9-10 |
| Synthesis of results | 21 | Present results of each meta-analysis done, including confidence intervals and measures of consistency. | 9-10 |
| Risk of bias across studies | 22 | Present results of any assessment of risk of bias across studies (see Item 15). | 8 |
| Additional analysis | 23 | Give results of additional analyses, if done (e.g., sensitivity or subgroup analyses, meta-regression [see Item 16]). | 9-10 |
| **DISCUSSION** | | |  |
| Summary of evidence | 24 | Summarize the main findings including the strength of evidence for each main outcome; consider their relevance to key groups (e.g., healthcare providers, users, and policy makers). | 10 |
| Limitations | 25 | Discuss limitations at study and outcome level (e.g., risk of bias), and at review-level (e.g., incomplete retrieval of identified research, reporting bias). | 11-12 |
| Conclusions | 26 | Provide a general interpretation of the results in the context of other evidence, and implications for future research. | 12 |
| **FUNDING** | | |  |
| Funding | 27 | Describe sources of funding for the systematic review and other support (e.g., supply of data); role of funders for the systematic review. | - |

## Table S1: Risk-of-bias assessment of included studies

| Study | Domain | | | | | | |  | Overall RoB Judgement |
| --- | --- | --- | --- | --- | --- | --- | --- | --- | --- |
|  | **Bias due to confounding** | **Bias in selection of participants** | **Bias in Measurement of Interventions** | **Bias due to departures from intended interventions** | **Bias due to missing data** | **Bias in measurement of outcomes** | **Bias in selection of reported results** |  |  |
| Drechshler et al, 2013 | Moderate | Moderate | Moderate | Moderate | Moderate | Low | Serious |  | Serious |
| Knobel et al, 2013 | Moderate | Serious | Moderate | Moderate | Moderate | Low | Serious |  | Serious |
| Krask et al, 2015 | Moderate | Moderate | Moderate | Moderate | Moderate | Low | Moderate |  | Moderate |
| Lang et al, 2015 | Moderate | Serious | Moderate | Moderate | Moderate | Low | Moderate |  | Serious |
| Moore et al, 2011 | Moderate | Serious | Moderate | Moderate | Moderate | Low | Moderate |  | Serious |
| Overton et al, 2013 | Moderate | Moderate | Moderate | Moderate | Moderate | Low | Moderate |  | Moderate |
| Rasmussen et al, 2015 | Moderate | Moderate | Moderate | Moderate | Moderate | Low | Moderate |  | Moderate |

## Table S2: Estimated pooled HRs from the sensitivity analyses

|  | **+/- 30% bias variance** | **+/- 50% bias variance** | **+/- 100% bias variance** | **Weighting by quality: 20% for low, 70% high** | **Weighting by quality: 50% for low, 80% high** | **Exclude low quality** | **Non-informative priors** |
| --- | --- | --- | --- | --- | --- | --- | --- |
| **Posterior mean** | 0.7 | 0.72 | 0.73 | 0.82 | 0.76 | 0.81 | 0.62 |
| **95% credible interval** | (0.46, 1.09) | (0.48, 1.10) | (0.47, 1.10) | (0.49, 1.35) | (0.50, 1.13) | (0.49, 1.37) | (0.35, 1.02) |
|  |  |  |  |  |  |  |  |
| ***I^2^*** | 0.56 | 0.51 | 0.49 | 0.49 | 0.43 | 0.45 | 0.6 |
| **Posterior probability HR<1** | 0.95 | 0.95 | 0.94 | 0.8 | 0.92 | 0.81 | 0.97 |
| **Posterior probability HR<0.9** | 0.9 | 0.87 | 0.86 | 0.66 | 0.81 | 0.69 | 0.94 |
| **Posterior probability HR<0.75** | 0.63 | 0.56 | 0.55 | 0.37 | 0.45 | 0.39 | 0.82 |
